# Neobavaisoflavone inhibits osteoclastogenesis through blocking RANKL signalling‐mediated TRAF6 and c‐Src recruitment and NF‐κB, MAPK and Akt pathways

**DOI:** 10.1111/jcmm.15543

**Published:** 2020-06-30

**Authors:** Huiwen Chen, Chao Fang, Xin Zhi, Shaojun Song, Yanqiu Gu, Xiaofei Chen, Jin Cui, Yan Hu, Weizong Weng, Qirong Zhou, Yajun Wang, Yao Wang, Hao Jiang, Xiaoqun Li, Liehu Cao, Xiao Chen, Jiacan Su

**Affiliations:** ^1^ Department of Orthopedics Trauma Shanghai Changhai Hospital Naval Military Medical University Yangpu District Shanghai China; ^2^ Basic Medical School Naval Military Medical University Yangpu District Shanghai China; ^3^ Department of Pharmacy Shanghai 9th People’s Hospital Huangpu District Shanghai China; ^4^ School of Pharmacy Second Military Medical University Yangpu District Shanghai China; ^5^ Department of Orthopedics Trauma Shanghai Luodian Hospital Baoshan District Shanghai China; ^6^ Department of Chemistry Fudan University Shanghai China; ^7^ China‐South Korea Bioengineering Center Jiading District Shanghai China

**Keywords:** c‐Src, neobavaisoflavone, osteoclastogenesis, osteoporosis, RANKL, TRAF6

## Abstract

*Psoralea corylifolia* (*P corylifolia*) has been popularly applied in traditional Chinese medicine decoction for treating osteoporosis and promoting fracture healing since centuries ago. However, the bioactive natural components remain unknown. In this study, applying comprehensive two‐dimensional cell membrane chromatographic/C18 column/time‐of‐flight mass spectrometry (2D CMC/C18 column/TOFMS) system, neobavaisoflavone (NBIF), for the first time, was identified for the bioaffinity with RAW 264.7 cells membranes from the extracts of *P corylifolia*. Here, we revealed that NBIF inhibited RANKL‐mediated osteoclastogenesis in bone marrow monocytes (BMMCs) and RAW264.7 cells dose dependently at the early stage. Moreover, NBIF inhibited osteoclasts function demonstrated by actin ring formation assay and pit‐formation assay. With regard to the underlying molecular mechanism, co‐immunoprecipitation showed that both the interactions of RANK with TRAF6 and with c‐Src were disrupted. In addition, NBIF inhibited the phosphorylation of P50, P65, IκB in NF‐κB pathway, ERK, JNK, P38 in MAPKs pathway, AKT in Akt pathway, accompanied with a blockade of calcium oscillation and inactivation of nuclear translocation of nuclear factor of activated T cells cytoplasmic 1 (NFATc1). In vivo, NBIF inhibited osteoclastogenesis, promoted osteogenesis and ameliorated bone loss in ovariectomized mice. In summary, *P corylifolia*‐derived NBIF inhibited RANKL‐mediated osteoclastogenesis by suppressing the recruitment of TRAF6 and c‐Src to RANK, inactivating NF‐κB, MAPKs, and Akt signalling pathways and inhibiting calcium oscillation and NFATc1 translocation. NBIF might serve as a promising candidate for the treatment of osteoclast‐associated osteopenic diseases.

## 1 INTRODUCTION

*P corylifolia* has been commonly used in herbal decoction based on the traditional Chinese medicine theory for its effectiveness in treating osteoporosis and promoting fracture healing. Abundant evidence has been emerging to support its efficacy for bone formation promotion and osteoporosis alleviation.^1^, ^2^ However, extracts from *P corylifolia* are bioactive mixtures and the specific effective monomers remain still by and large unknown. Figuring out potential bioactive products would pave way for the exploration of novel drugs for bone metabolism disorders.

Currently, a comprehensive two‐dimensional cell membrane chromatographic/C18 column/time‐of‐flight mass spectrometry (2D CMC/C18 column/TOFMS) system analysis system has been developed for screening potential bioactive components from Chinese herbal medicines, based on the binding or bioaffinity of unidentified components to their membrane targets.^3^, ^4^ In our study, ethanol extracts from the seeds of *P corylifolia* were screened by 2D RAW 264.7 cells CMC/C18 column/TOFMS system, and neobavaisoflavone (NBIF) was identified as a potentially bioactive chemical component binding to the membrane of RAW 264.7 cells.

Bone metabolism homeostasis relies on the duel competitive role of osteoblasts and osteoclasts.^5^ Disturbance of this delicate balance as a result of excessive bone resorption by overactivated osteoclasts contributes to the occurrence of various metabolic bone diseases, like post‐menopausal osteoporosis (PMOP) and rheumatoid arthritis (RA).^6^ Therefore, inhibiting overactivated osteoclastogenesis could be an effective strategy to find a cure for pathological bone loss in these diseases.^7^, ^8^, ^9^, ^10^ Osteoclasts originate from the haematopoietic cell line and differentiate from bone marrow monocytes that are stimulated by two indispensable cytokines, macrophage colony‐stimulating factor (M‐CSF) and receptor activator of nuclear factor‐κB ligand (RANKL).^11^, ^12^ The M‐CSF binding to c‐Fms keeps the survival and proliferation of BMMCs and pre‐osteoclasts and initiates BMMCs differentiation into osteoclast precursors, while the conjunct of RANKL and RANK leads to terminal differentiation into mature osteoclasts.^13^ RAW 264.7 cells, a widely used mouse monocytic cell line, express RANK and have been shown to differentiate into functional osteoclasts upon recombinant RANKL stimulation.^14^ In addition, the binding of RANKL to RANK recruits tumour necrosis factor receptor‐associated factors (TRAFs), of which TRAF6 is the most important one.^15^ It is worth noting that c‐Src is also recruited by activated RANK to organize osteoclast's cytoskeleton and function.^16^ As a result, several downstream pathways are subsequently tranduced by the activated RANK‐TRAF6 complex and RANK‐c‐Src conjunct. The well‐recognized downstream signalling pathways involving in this cascade include NF‐κB (IκB, P50, P52, Rel A, RelB, c‐Rel) and MAPKs (ERK, JNK, P38) activated by TRAF6 recruitments, and Akt induced by c‐Src recruitments.^17^, ^18^, ^19^ RANKL‐RANK interactions induced activation of Akt signalling pathway also triggers cytoplasmic calcium released by calcium oscillation, which ultimately increases the expression and translocation of NFATc1.^20^, ^21^, ^22^ NFATc1 is the core transcriptional factor in the differentiation and maturation of osteoclasts, and it dominates the expression of multiple osteoclastogenesis‐related genes, including tartrate‐resistant acid phosphatase (TRAP), matrix metalloproteinase (MMP)‐9, cathepsin K, calcitonin receptor (CTR), all of which are responsible for the terminal function of osteoclasts.^23^, ^24^, ^25^, ^26^

Recently, NBIF, an isoflavonoid originally isolated from the seeds of *P corylifolia*, has attracted much attention because of its anti‐inflammation, anti‐cancer and anti‐oxidation properties.^27^, ^28^ Inflammation plays a pivotal role in both osteoclastogenesis and osteoporosis.^29^ A recent study reported that NBIF inhibits inflammatory mediators in activated RAW264.7 macrophages.^30^ Intriguingly, NBIF was previously reported to stimulate osteogenesis in vitro by P38 MAPK pathway.^31^ However, the role of NBIF in osteoclastogenesis remains unclear. Here in our report, we demonstrated that NBIF might serve as an effective osteoclastogenesis inhibitor and an osteogenic promotor, ameliorate ovariectomy‐induced bone loss in mice.

## 2 MATERIALS AND METHODS

### 2.1 Reagents and animals

*Psoralea corylifolia* was purchased from Zhongda TCM Store (Shanghai, China). NBIF (purity ≥98%) was ordered from Shidande Company (Shanghai, China, http://www.nature‐standard.com/product/html/1138.html). RAW 264.7 cells were supplied by Shanghai Institutes for Biological Sciences (Shanghai, China). Alpha‐modified minimal essential medium (α‐MEM) and foetal bovine serum (FBS) were obtained from Hyclone (Logan, UT, USA). Murine M‐CSF and RANKL were ordered from R&D Systems (MN, USA). Cell Counting Kit‐8 (CCK8), Tartrate‐Resistant Acid Phosphatase (TRAP) staining kit, Alkaline Phosphatase (ALP) staining kit and Alizarin Red staining kit were provided by Sigma‐Aldrich (St. Louis, MO, USA). C57BL/6 female mice (8‐week‐old, weight 20‐25 g) were ordered from Slaccas (Shanghai, China).

### 2.2 Preparation of samples and RAW264.7‐CMC column

Firstly, *Psoralea corylifolia* was smashed into powder by a pulverizer. Then, the powder was mixed with 60% ethanol at 60‐80°C water bath along with ultrasonic fragmentation for 2 hours. The ethanol extract was condensed by the rotary evaporator to 1 g/mL. After that, the ethanol extract was filtered by 0.2 μm filter membrane and stocked at 4°C for further use.

For RAW264.7 cell membrane preparation, 3.5 × 10^7^ RAW264.7 cells were harvested and washed by PBS for 3 times and centrifuged at 110 × g for 10 minutes. Then, PBS was added to suspend cells, after which disrupted by an ultrasonic processor of 3 cycles (3 seconds for 400 W and 15 seconds for internal each cycle). The homogenate was then subjected to centrifugation at 1000 × g for 10 minutes. The supernatant was collected for further centrifugation at 12 000 × g for 20 minutes. The precipitation was collected and suspended in 5 mL PBS, which was regarded as cell membrane suspension.

For RAW264.7 cell membrane stationary phase (CMSP) preparation, 0.04 g activated silica under vacuum and agitation condition was mixed with RAW264.7 cell membrane suspension to absorb cell membrane. After incubation at 4°C overnight, the CMSP was washed by PBS for 3 times, centrifuging at 110 × g for 5 minutes. The pellet was collected, suspended in PBS and put into a column. The flow rate of packing was controlled by a linear program, and the equilibrated flow rate is 0.2 mL/min at 37°C. Then, the column was stored in PBS at 4°C.

### 2.3 Identification of the bioactive compound from *P Corylifolia*

As previously described,^3^, ^32^ a comprehensive 2D RAW 264.7 cells CMC/C18 column/TOFMS system was used to detect the affinity between bioactive mixtures from *P Corylifolia* and the cell membrane of RAW 264.7. Briefly, at position 1, the first retention fraction recognized by RAW 264.7/CMC column model was enriched into the Enriched Column 1 (EC1). After the 10‐port valve switched to position 2, the enriched components were pumped into the C18 analytical column and TOF‐MS for qualitative analysis. Simultaneously, the second retention fraction was pumped into the Enriched Column 2 (EC2) and then went through a second dimension analysis. Next, the screened bioactive compounds were identified by resorting to the Traditional Chinese Medicine and Chemical Composition Database by molecular formula comparison. (http://202.127.145.134/scdb/main/tcm_introduce.asp).

### 2.4 Animal experimental designs

All animal experiments were conducted following the standards of Bioethics Committee in Changhai Hospital (SYXK 2015‐0017). Mice were randomly distributed to 3 groups (5 mice in each group): sham group, OVX group and NBIF group. Mice in the OVX group were treated with normal saline, while mice in the NBIF group were treated with NBIF. All mice were anaesthetized with 5% chloral hydrate. Ovaries were merely exposed from the surrounding adipose tissue in the sham group, and bilateral ovaries were removed in both OVX group and the NBIF group. After 1 day for post‐operative recovery, OVX group and NBIF group were received intraperitoneal (i.p.) injection with normal saline and NBIF (30 mg/kg), respectively. Six weeks later, all groups were killed by an overdose of chloral hydrate, after which bilateral femurs and blood were collected for further measurements.

### 2.5 Cytotoxicity assays

The CCK‐8 assay was executed according to the standard protocols. BMMCs and RAW264.7 cells were cultured and seeded on the 96‐well plate (1 × 10^4^ cells/ well) for 24 hours incubation. Then, BMMCs were cultured in induction culture medium containing RANKL (50 ng/mL) with M‐CSF (30 ng/mL), while RAW264.7 cells with RANKL (50 ng/mL) and without M‐CSF, after which the cells were subjected to various concentrations of NBIF (0, 2, 4, 8, 16, 32 and 64 μM) for 48 hours. Thereafter, CCK‐8 solution (Sigma) was added to each well, incubating for another 2 hours for absorbance evaluation. Microplate reader was used to measure the absorbance at 490 nm.

### 2.6 Osteoclastogenesis assays

BMMCs were separated from the bilateral femurs of mice. RAW 264.7 cells and BMMCs were then seeded on 96‐well plates (1.5 × 10^4^ cells/well) in α‐MEM media with 10% FBS, 1% streptomycin and penicillin mixture. M‐CSF (30 ng/mL) and RANKL (50 ng/mL) were used to stimulate osteoclast differentiation. Cells of the third passage were assigned to one control group and four groups intervened with ascending levels of NBIF concentrations (0, 2, 4 and 8 μM) for 7 days. Then, TRAP staining was performed on these groups of cells in accordance with the general protocols. Multinucleated (more than 5 nucleus) TRAP‐positive cells were considered as osteoclasts, and the number was counted.

### 2.7 Osteogenesis assays

Bone marrow mesenchymal stromal cells (BMSCs) were separated by flushing the bone marrow out of femurs and tibias from 4‐week‐old C57/BL6 male mice. To induce osteogenesis differentiation, cells were cultured in α‐MEM supplemented with 10% FBS, 10^−8^ mol/L dexamethasone, 10 mM β‐glycerophosphate and 50 mg/mL ascorbic acid. Differentiated cells were fixed and stained with alkaline phosphatase (ALP) staining at day 7 and stained with Alizarin Red staining at day 21.

### 2.8 F‐actin staining

For fibrous actin (F‐actin) staining, RAW264.7 cells in 96‐well plates (1.5 × 10^4^ cells/well) induced by M‐CSF (30 ng/mL) and RANKL (50 ng/mL) for 7 days were fixed with 3.75% formaldehyde in cold PBS for 15 minutes. After that, cells were subject to 0.5% Triton X‐100 for 3‐minute permeabilization followed by non‐fat dry milk used for a blockade of non‐specific binding. Then, permeabilized cells were incubated in rhodamine‐conjugated dilution with 1% bovine serum albumin (BSA) for 20 minutes at room temperature. Before being photographed, cells were counterstained by DAPI for 10 minutes. The number of F‐actin rings and the number of osteoclasts were counted for further statistical analysis.

### 2.9 Pit‐formation assays

RAW264.7 cells (1.5 × 10^4^ cells/well) were induced in the presence or absence of RANKL for 7 days. Following collagenase II (Sigma‐Aldrich) digestion, cells were then harvested and seeded onto the plates coated with bone biomimetic synthetic surface (Corning, St. Lowell, MA, USA), subject to ascending level of NBIF concentrations (0, 2, 4 and 8 μM). Two days later, cell remains on bone biomimetic synthetic surface were cleaned up and the surface was rinsed by swirling in distilled water and subjected to air dry afterward. Pit‐formation areas on the biomimetic plate surface were captured by Light Microscope (OLYMPUS‐BX53) and measured by Image J software.

### 2.10 Microcomputed tomography

Femurs isolated from mice were fixed in 4% paraformaldehyde (PFA). Micro‐CT (Skyscan, Antwerp, Belgium) was used to scan each distal femoral metaphysis. The analysis conditions met the following parameters: the voltage was 80 kV, the electric current was 124 μA and the resolution was 8 μm. Then, scans were integrated into 2D and 3D images. Quantitative data of femur parameters were obtained as follows: bone surface/tissue volume (BS/TV), bone volume/tissue volume (BV/TV), trabecular bone number (Tb.N) and bone mineral density (BMD). These parameters were calculated using the built‐in software.

### 2.11 Histological analysis

Femurs were isolated, fixed in 4% PFA for 48 hours and decalcified for 2 weeks. Two weeks later, these femurs were embedded in paraffin, and then, 4 μm thick sections were cut into slices by the microtome and stained by haematoxylin and eosin (H&E), TRAP staining kit and stained for Osteocalcin (OCN). Light microscope (OLYMPUS‐BX53) was used to observe and photograph the femur trabecular area. TRAP‐positive multinucleated osteoclasts with 3 or more nuclei were calculated.

### 2.12 Serum biochemistry analysis

Blood was sampled from mouse eyes. Sera were then collected after 1000 *g* centrifugation for 15 minutes. According to the general instructions, ELISA kit was employed for biochemical detection of serum carboxy‐terminal telopeptide of type‐l collagen (CTX‐1), and Tartrate‐resistant acid phosphatase 5b (TRAcp5B) (IDS plc, Boldon, UK), OCN (Immutopics, San Diego, CA, USA).

### 2.13 Immunofluorescence assays

For RANK and c‐Fms immunofluorescence staining, BMMCs cells were isolated and seeded on the 6‐well plates at a density of 1.0 × 10^5^ cells/well, induced by 30 ng/mL M‐CSF and incubated with 8 μM NBIF for 24 hours. For P65 or NFATc1 immunofluorescence staining, RAW264.7 cells were cultured on the 6‐well plates (1.0 × 10^5^ cells/well) and then stimulated by 30 ng/mL M‐CSF and 50 ng/mL RANKL which was followed by 1‐hour NBIF treatments (8 μM). After incubation for the indicated time‐point, 4% PFA was used to fix cells for 10 minutes and washed with 1× PBS for three times, after which cells were permeabilized by 0.1% Triton X‐100 for 5 minutes and blocked with 5% BSA in PBS for 30 minutes. Cells were added with anti‐RANK antibody (1:100), anti‐c‐Fms antibody (1:200), anti‐P65 antibody (2 μg/mL) or anti‐NFATc1 antibody (1:20), respectively, and then incubated at 4 degree Celsius overnight. FITC‐conjugated and Cy3‐conjugated secondary antibodies (1:200) followed, and cells were stained with Hoechst 33258 or DAPI (Sigma, St. Louis, MO, USA) to visualize cell nuclei. Images were obtained using confocal laser scanning microscopy (Olympus, Tokyo, Japan).

### 2.14 Cytoplasmic Ca^2+^ measurement

BMMCs cells were isolated and cultured on the 48‐well plates (2 × 10^4^ cell/well) with M‐CSF (50 ng/mL) for 24 hours. Then, cells were induced in the presence of M‐CSF (50 ng/mL) with or without RANKL (50 ng/mL) and treated with NBIF (8 μM) for the next 48 hours. After washing with assay buffer, cells were added with 4 μM Fluo4 staining solution. The cytoplasmic Ca^2+^ was examined by inverted fluorescence microscope (Nikon Ti‐U) at 488 nm, and the results were analysed using Nikon Basic Research Software. After scanned and plotted at the interval of 2 seconds for 3 minutes, cell images recorded with 2 or more peaks were recorded as oscillating. Moreover, the fluorescence intensities between the highest and lowest group within the oscillating area were detected.

### 2.15 Quantitative real‐time PCR

The whole RNA was extracted with TRIzol reagent (Invitrogen, Carlsbad, CA, USA). Next, cDNA was inversely transcribed from RNA samples (Invitrogen, San Diego, CA, USA) according to standard instructions. RT‐PCR was performed by ABI ViiA7 Real‐Time System (Applied Biosystems, Foster City, CA, USA). Primers were prepared as follows: RANK forward primer (5′‐ATCCAGCAGGGAAGCAAA‐3′) reverse primer (5′‐GGGACACGGGCATAGAGT‐3′), c‐Fms forward primer (5′‐TTGCCTTTGAATTTGTAGACC‐3′) reverse primer (5′‐CTCGGTGGCGTTAGCATT‐3′), β‐actin forward primer (5′‐GTCCCTCACCCTCCCAAAAG‐3′) reverse primer (5′‐GCTGCCTCAACACCTCAACCC‐3′).

### 2.16 Western blot

Western blot was used to detect the expression of osteoclastogenesis‐associated markers genes (TRAP, CTR, MMP‐9 and Cathepsin K). Briefly, RAW264.7 cells were cultured in 6‐well plates (1 × 10^5^ cell/well), subsequently induced with or without RANKL (50 ng/mL), in the presence of different NBIF concentrations (0, 2, 4 and 8 μM). Cells were harvested for Western blot analysis at day 7 after the induction. For the detection of phosphorylation of IκB, P65, P50, ERK, JNK, P38 and Akt, the cells were pre‐incubated in NBIF (0, 8 μM) for 1 hour and then treated with or without RANKL (50 ng/mL) for 10‐15 minutes before they were harvested. Proteins were prepared and quantified with the BCA protein kit (Thermo Fisher, Waltham, MA, USA), resolved in SDS‐PAGE, electrotransferred to the membrane and blocked by the mixture of Tris‐buffered saline and tween (TBST) in 5% skim milk. After the incubation with primary antibodies overnight (4°C) and anti‐rabbit HRP conjugated secondary antibodies, images were visualized by the chemiluminescence. Image J software was used to quantify the protein band intensities. Primary antibodies against specific proteins (IκB 1:200, p‐IκB 1:10 000, P65 1:2000, p‐P65 1:2000, P50 1:2000, p‐P50 1:200, ERK 1:10 000, p‐ERK 1:1000, JNK 1:1000, p‐JNK 1:2000, P38 1:2000, p‐P38 1:1000, Akt 1:10 000, p‐Akt 1:1000, β‐actin 1:2000, TRAP 1:2000, CTR 1:1000, MMP‐9 1:1000, Cathepsin K 1:2000) were provided by Abcam (Cambridge, UK). Secondary antibodies were antimouse/rabbit antibodies supplied by Santa Cruz (Dallas, TX, USA).

### 2.17 Co‐immunoprecipitation (Co‐IP) assay

RAW264.7 cells were treated with NBIF and then underwent homogenization and centrifugation; the supernatant was collected and incubated with beads bounded to the specific (TRAF6 or c‐Src) antibody. The mixture was then centrifuged with the supernatant discarded. The beads were washed with washing buffer to collect the protein complex. Subsequently, sulphate‐polyacrylamide gel electrophoresis (SDS‐PAGE) and Western blot analysis were followed. The antibodies were used as follows: anti‐TRAF6 antibody (Abcam), anti‐c‐Src antibody (Abcam) and anti‐β‐actin antibody (Abcam).

### 2.18 Statistical analysis

Total results were presented as mean ± SD. Two‐tailed non‐paired Student's *t* test statistical analysis was performed to compare 2 groups, and 3 or more groups were compared by one‐way ANOVA. GraphPad Prism 5 software was employed to undertake statistical analyses. *P < 0.05, **P < 0.01 or ***P < 0.001 were presented as different levels of statistical significance.

## 3 RESULTS

### 3.1 NBIF is a bioactive compound extracted from *P corylifolia*

Following a novel strategy to identify potential active components from interested traditional Chinese herbs,^3^ we finally discovered that NBIF was a potential bioactive compound which could be extracted from ***P corylifolia***.

**Figure 1:**
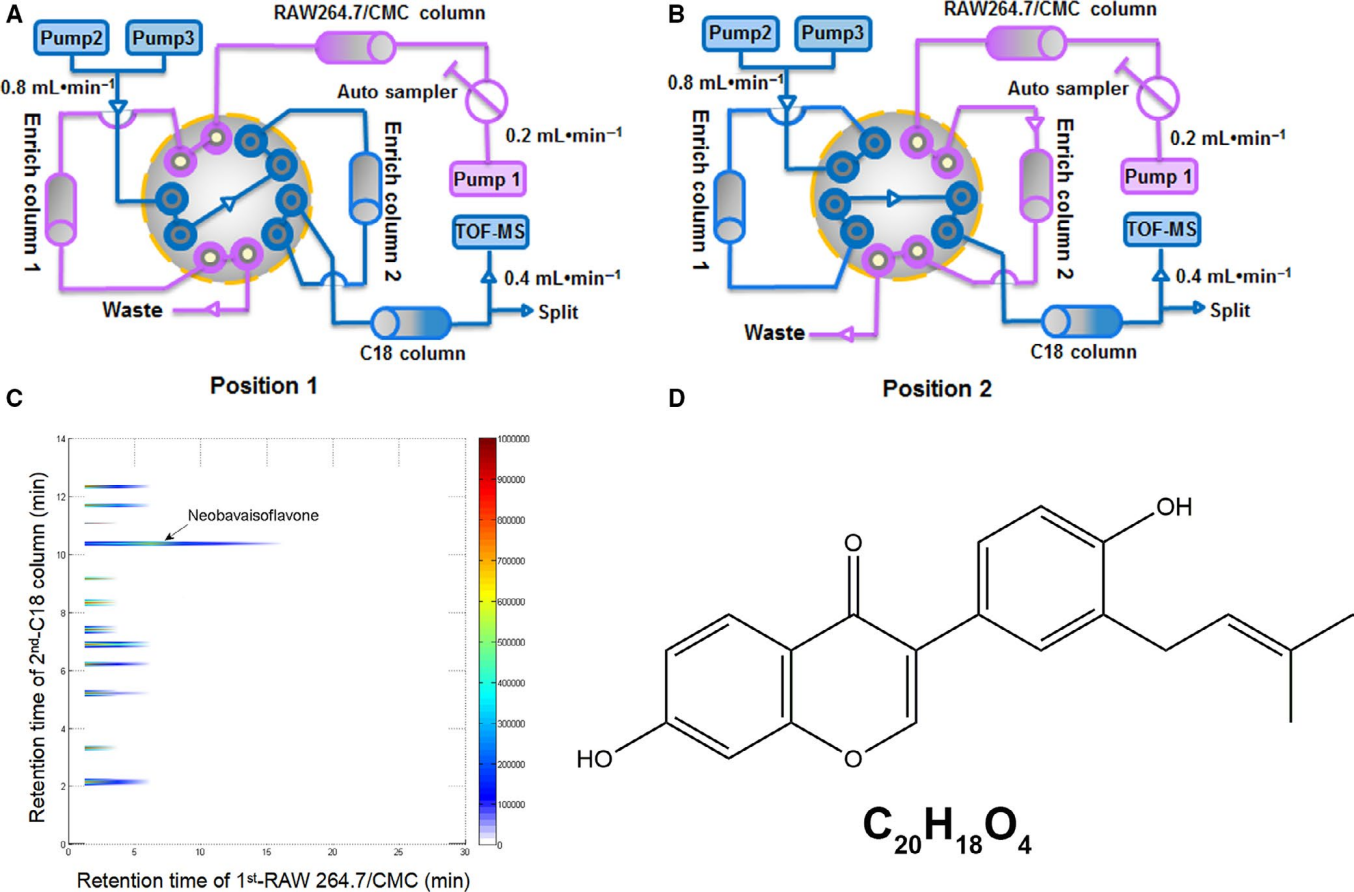
Screening bioactive components from prepared extracts of *P Corylifolia*. A and B, Brief scheme of the comprehensive 2D RAW264.7 cells CMC/C18 column/TOFMS system. Position 1 (A) and position 2 (B). C, Typical 2D plots of *P Corylifolia* extracts obtain by 2D RAW264.7 cells CMC/C18 column/TOFMS system. D, Chemical structure of NBIF

As mentioned above in the Methods, using 2D RAW264.7 cells/CMC/C18 column/TOFMS system (Figure 1A‐B), we discovered that only one component extracted from the seeds of *P corylifolia* displayed a fine affinity with RAW 264.7 cell membrane, represented by strong retention behaviour and the peak reaching time at about 5 minutes (Figure 1C). We identified that the molecular formula of this compound was C_20_H_18_O_4_, and then, we searched and compared the components of *P corylifolia* on the Traditional Chinese Medicine Integrated Database. Finally, we confirmed that this bioactive ingredient was NBIF (Figure 1D). Moreover, through literature research, we found no publication reporting the efficacy of NBIF related to osteoclastogenesis. Hence, we focused on NBIF in our following work.

### 3.2 NBIF suppressed osteoclastogenesis in vitro

**Figure 2:**
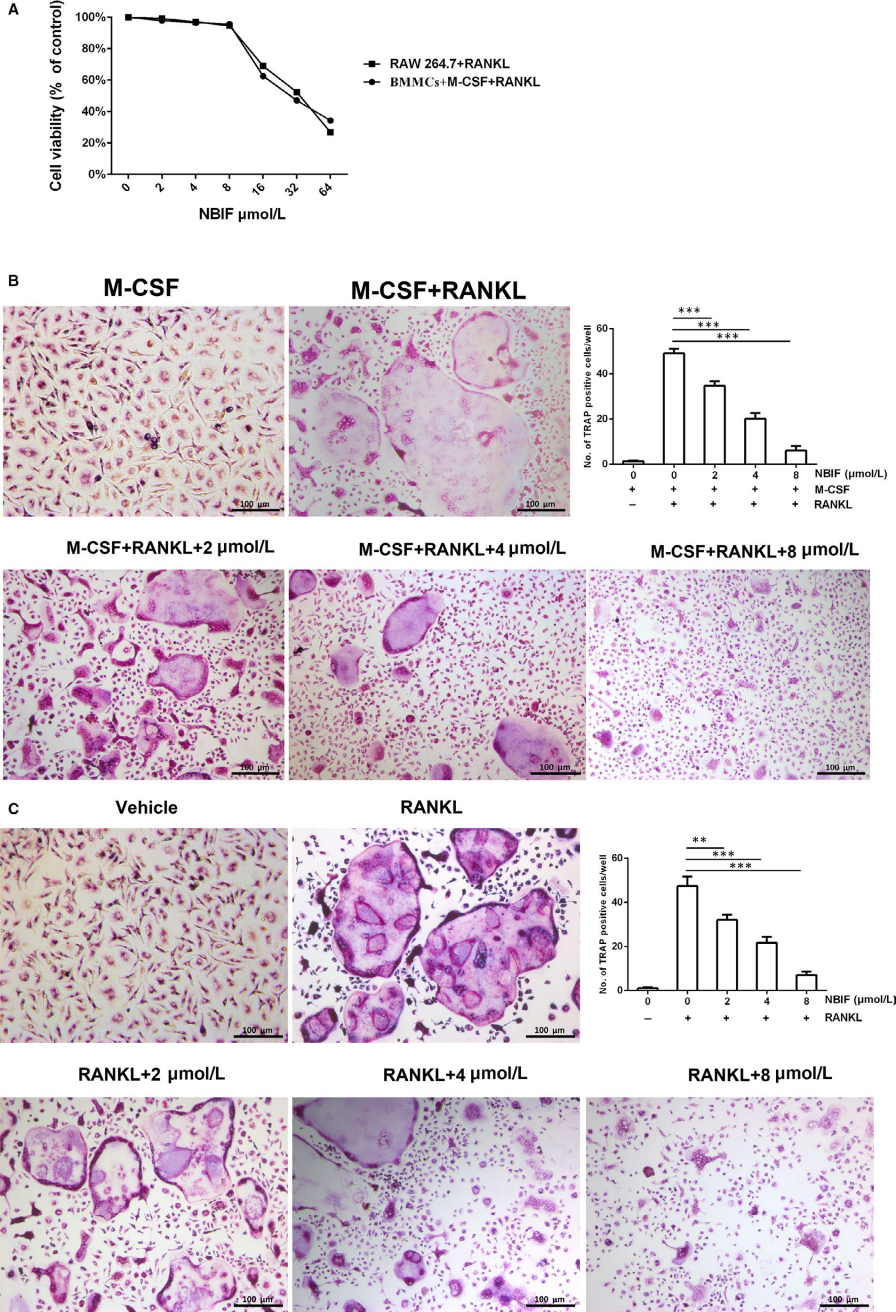
NBIF inhibits osteoclastogenesis in vitro. A, CCK‐8 analysis of NBIF’s cytotoxicity in RAW264.7 cells and BMMCs. Experiments were repeated 3 times. B, Formation of TRAP‐positive cells from RANKL‐induced BMMCs, treated with different levels of NBIF concentrations (0, 2, 4 and 8 μM) for 7 days, and quantification of osteoclasts. C, Formation of TRAP‐positive cells from RANKL‐induced RAW264.7 cells, treated with different levels of NBIF concentrations (0, 2, 4 and 8 μM) for 7 days, and quantification of osteoclasts. (**P < .01, ***P < .001 vs. RANKL group)

The cytotoxic effect of NBIF (0, 2, 4, 8, 16, 32 and 64 μM) on RANKL‐induced BMMCs and RAW264.7 cells was confirmed by CCK‐8 assay. The IC50 value for NBIF cytotoxicity on BMMCs was 31.94 μM, and the IC50 value for NBIF cytotoxicity on RAW264.7 cells was 32.54 μM. The CCK‐8 assay results showed no significant cytotoxicity of NBIF below the concentration of 8 μM (Figure 2A).

To further assess the effects of NBIF on osteoclast formation and function, RANKL‐induced osteoclastogenesis assay was carried out in BMMCs and RAW264.7 cells, which were exposed to various concentrations of NBIF (0, 2, 4 and 8 μM). TRAP‐positive multinucleated cells were counted to show the osteoclast differentiation induced by RANKL. As the results presented, the number of TRAP‐positive multinucleated cells showed a dramatic decrease when treating with NBIF in a dose‐dependent way (Figure 2B and C).

### 3.3 NBIF inhibited the function of osteoclasts in vitro

**Figure 3:**
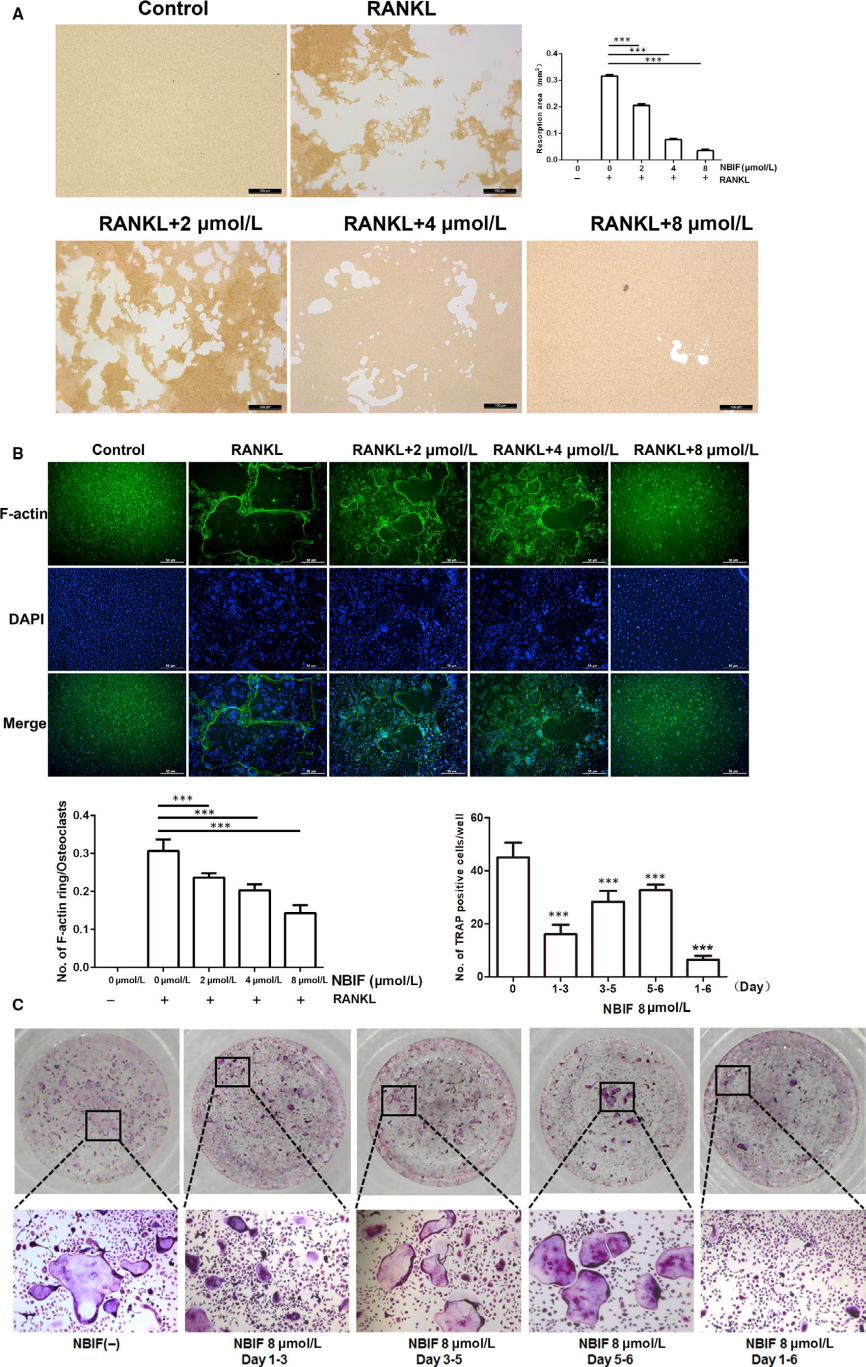
NBIF inhibits osteoclast function in vitro and suppressed osteoclastogenesis at the early phase. A, Representative images of pit formation by osteoclasts derived from RANKL‐induced RAW264.7 with or without different levels of NBIF concentrations (0, 2, 4 and 8 μM) and quantification of pits area. B, Representative immunofluorescent images of actin ring structures of osteoclasts derived from RANKL‐induced RAW264.7 different levels of NBIF concentrations (0, 2, 4 and 8 μM) and the ratio of the actin ring numbers compared with total osteoclast numbers. C, Representative images of the formation of TRAP‐positive cells treated with 8 μM NBIF (Group 1: without NBIF) at different time‐points and durations (Group 2 to 5: day 1‐3, day 3‐5, day 5‐6 and day 1‐6, respectively). Data are presented as mean ± SEM (n = 3) (*P < .05, ***P < .001)

Pit‐formation assay and F‐actin rings formation assay were carried out to detect whether osteoclasts’ functions were impaired. Pit‐formation assay showed that bone resorption activity was dose‐dependently attenuated by NBIF (Figure 3A). Furthermore, F‐actin rings were regarded as the characteristic structure of mature osteoclasts. To further determine the inhibitory effect of NBIF on osteoclast function, immunofluorescence staining of F‐actin was performed in RAW264.7 cells which were exposed to NBIF treatments. Similar to the prior inhibitory effects, it showed that NBIF significantly inhibited the formation of F‐actin ring structures, demonstrated by the decrement of F‐actin numbers compared to total osteoclast numbers (Figure 3B). In brief, the results showed that the function of osteoclasts could be notably impaired by NBIF.

### 3.4 NBIF inhibited osteoclastogenesis at the early phase

To further identify at which specific phase NBIF displayed its inhibitory effect on osteoclastogenesis, RANKL‐induced RAW264.7 cells were incubated with NBIF (8 μM) at different time‐points and durations (day 1‐3, day 3‐5, day 5‐6 and day 1‐6). TRAP staining illustrated that osteoclast differentiation was more strongly inhibited by NBIF treatments in the first several days than that in the later days, implying that NBIF mainly suppressed osteoclast differentiation at the early phase (Figure 3C).

### 3.5 NBIF showed little effects on M‐CSF‐stimulated BMMCs proliferation

Our CCK‐8 assay results showed that BMMCs proliferation induced by M‐CSF was not significantly influenced by NBIF (below 8 μM), indicating that NBIF exerted little influence on BMMCs proliferation (Figure S1).

### 3.6 NBIF showed little influence on RANK and c‐Fms expressions during osteoclastogenesis

To assess whether NBIF influenced RANK (receptor of RANKL) and c‐Fms (receptor of M‐CSF), RT‐PCR was employed to detect the transcriptional levels of RANK and c‐Fms after the induction of M‐CSF (30 ng/mL). The results revealed that the transcriptional level of RANK was up‐regulated by M‐CSF incubation while NBIF showed no influence to reverse this effect. Neither M‐CSF nor NBIF altered the expression of c‐Fms (Figure S2A). Similar to the findings of RT‐PCR, immunofluorescence assays confirmed that RANK and c‐Fms fluorescent intensities were not significantly up‐regulated or down‐regulated when exposed to NBIF (Figure S2B). To sum up, these results implied that NBIF had no effect on RANK and c‐Fms during osteoclast formation.

### 3.7 NBIF exerted osteogenic effects on the osteoblastic differentiation of bone marrow mesenchymal stem cells (BMSCs)

To confirm the reported osteogenic effects of NBIF, we performed ALP, Alizarin Red staining after the osteogenic induction with or without NBIF (1 μM). Consistent with the published data,^31^ ALP staining and Alizarin Red staining indicated that NBIF stimulated osteogenesis in BMSCs in vitro (Figure S3). Thus, NBIF might serve as both as osteoclastogenesis inhibitor and osteogenic promotor at the same time.

### 3.8 NBIF inhibited RANKL‐induced NF‐κB and MAPKs pathways activation and interrupted RANK‐TRAF6 interaction in osteoclastogenesis

**Figure 4:**
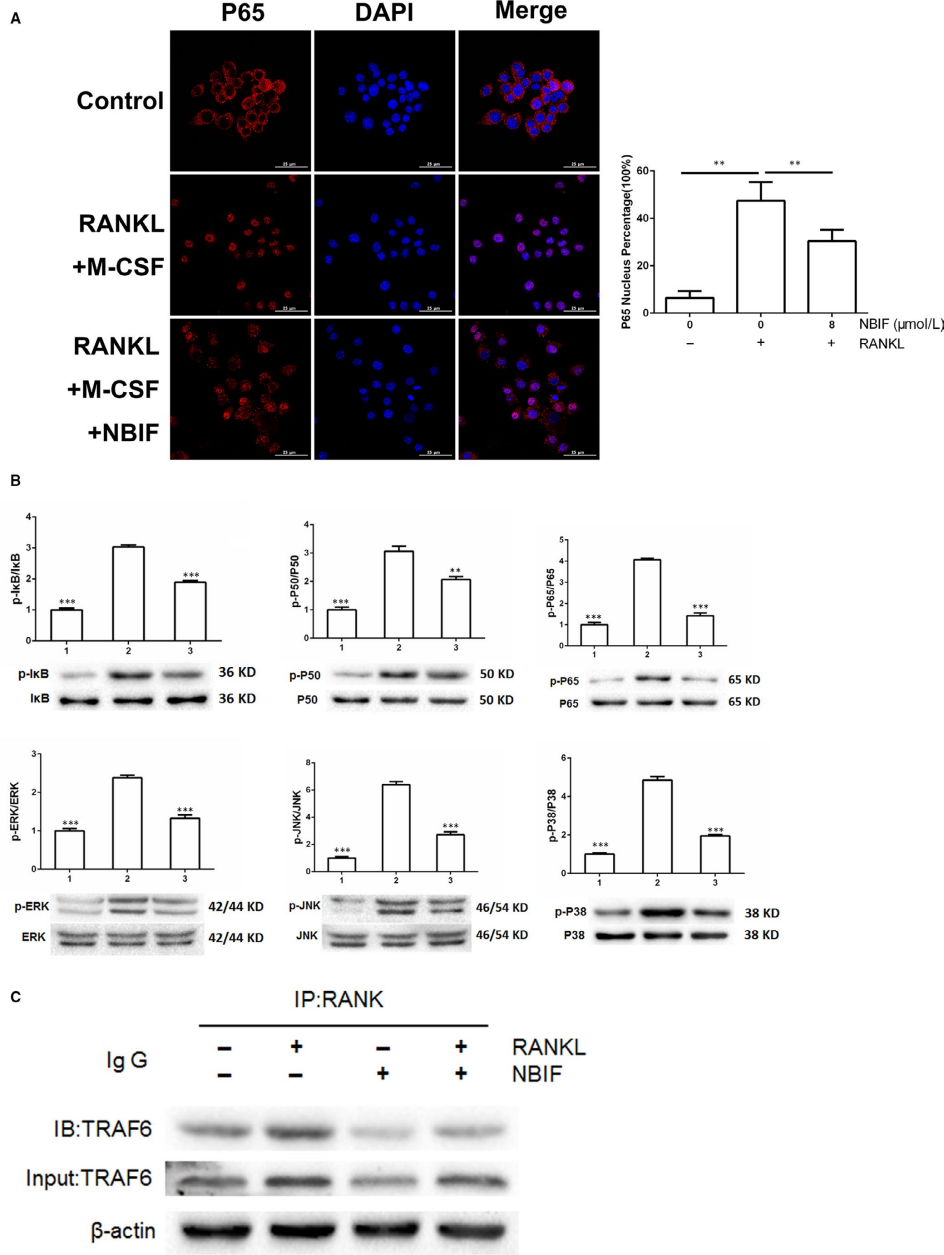
NBIF inhibits RANKL‐induced activation of NF‐κB, MAPKs signalling pathway during osteoclastogenesis and disrupts the interactions between RANK and TRAF6. A, Representative immunofluorescent staining images and quantification of the nuclear translocation of P65 of induced RAW 264.7 cells treated with or without 8 μM NBIF. B, Representative images and quantification of the phosphorylation of the members of the NF‐κB pathway (IκB, P50 and P65), phosphorylation of the members of MAPKs (ERK, JNK and P38) by Western blot assay. Groups are Group 1: RAW 264.7 cells without induction; Group 2: RAW264.7 cells induced by RANKL treated with PBS. Group 3: RAW264.7 cells induced by RANKL treated with NBIF (8 μM). ***P < .001 vs. Group 2. Data are presented as mean ± SEM (n = 3). C, Representative images of the RANK‐TRAF6 interactions illustrated by co‐immunoprecipitation images of RAW 264.7 cells with or without the treatment of NBIF (8 μM)

The activation of RANKL‐stimulated NF‐κB pathway is essential for osteoclast differentiation. The induction of RANKL stimulated the nuclear translocation of P65 from cytoplasm to nucleus, and this nuclear translocation is programmed to follow the phosphorylation of Inhibitor of Kappa B (IκB), P65 and P50. To examine the role of NBIF in NF‐κB pathway activation, immunofluorescence staining P65 was performed in RANKL‐induced RAW 264.7cells. It founded that the nuclear translocation of P65 was notably blocked, and P65 was confined in the cytoplasm by the treatments of NBIF, implying an impaired NF‐κB pathway during the osteoclastogenetic process (Figure 4A). Next, Western blot assay was employed to detect whether NBIF exerted any inhibitory impact on the RANKL‐induced phosphorylation of IκB, P65 and P50. Western blot assay revealed that the expressions of these aforementioned specific downstream signals of the NF‐κB pathway were remarkably suppressed by NBIF treatments. Moreover, besides the NF‐κB pathway, the MAPKs pathway is equally indispensable during osteoclastogenesis. Western blot showed that, after RANKL induction, the phosphorylation levels of MAPKs (ERK, JNK and P38) were similarly attenuated by NBIF intervention (Figure 4B). To further explore the down‐regulating mechanism of NF‐κB and MAPKs pathways, Co‐IP was carried out to identify the role of NBIF in TRAF6 recruitment by RANK activation after the stimulation of RANKL in RAW264.7 cells. The results indicated that TRAF6 was recruited and bonded with RANK after RANKL stimulation, whereas NBIF notably blocked this process, suggesting that RANKL‐induced recruitment of TRAF6 was impaired when exposed to NBIF (Figure 4C). Taken together, the above findings suggested that NBIF RANKL‐induced NF‐κB, MAPKs pathways activation and abrogated the interaction of RANK and TRAF6 during osteoclastogenesis.

### 3.9 NBIF inhibited RANKL‐induced Akt pathway activation and blocked RANK‐c‐Src interaction

**Figure 5:**
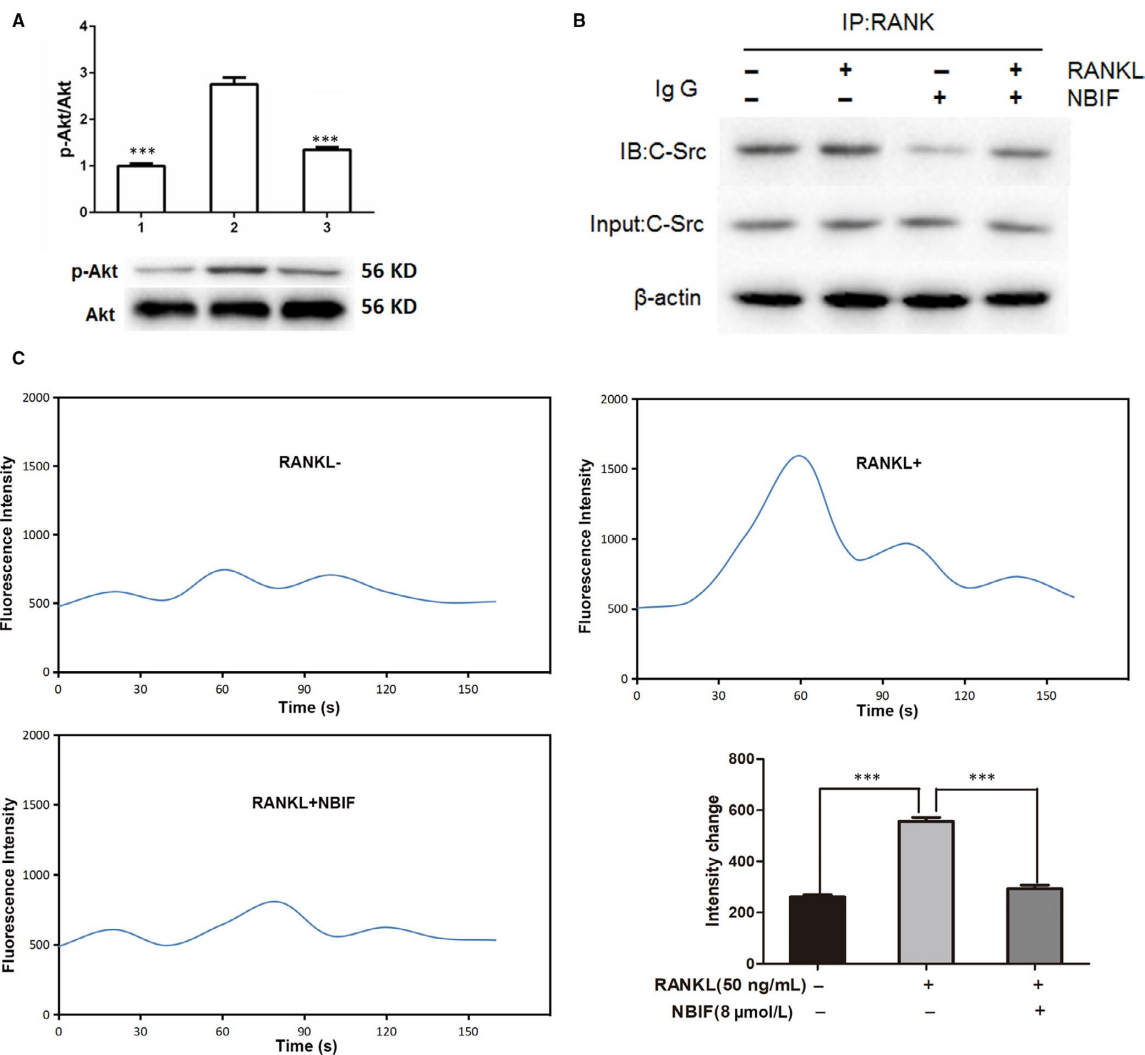
NBIF inhibits RANKL‐induced activation of Akt signalling pathway during osteoclastogenesis, blocks RANK‐c‐Src interactions and inactivates calcium oscillation during osteoclastogenesis. Groups are Group 1: RAW 264.7 cells without induction; Group 2: RAW264.7 cells induced by RANKL treated with PBS. Group 3: RAW264.7 cells induced by RANKL treated with NBIF (8 μM). A, Representative images and quantification of the phosphorylation of Akt during osteoclastogenesis by Western blot assay. B, NBIF inhibits RANK‐c‐Src interactions. C, Representative images of Ca^2+^ oscillation and quantitative analysis fluorescence intensity change in RAW 264.7 cells with or without RANKL (50 ng/mL) and NBIF (8 μM). Data are presented as mean ± SEM (n = 3). ***P < .001 vs. Group 2

Activation of the Akt pathway stimulated by RANKL also plays a crucial role in the process of osteoclastogenesis. Western blot revealed that the phosphorylation of Akt was significantly elevated by the induction of RANKL, whereas remarkably down‐regulated by NBIF treatments (Figure 5A). Moreover, Co‐IP was employed to determine whether NBIF disrupted the interaction between RANK and c‐Src stimulated by RANKL incubation. Co‐IP displayed that c‐Src effectively bound to RANK after RANKL induction, while the recruitment was disrupted by the treatment of NBIF (Figure 5B). Ca^2+^ oscillation is also provoked by Akt signalling activation and serves as a vital messenger for signalling transduction of osteoclast differentiation. Here, the fluctuation of the Ca^2+^ level was detected to further identify the role of NBIF in calcium signalling. The results showed that RANKL induction stimulated cytoplasmic Ca^2+^ release, while this effect was significantly diminished by NBIF intervention (Figure 5C).

### 3.10 NBIF suppressed translocation of NFATc1 and osteoclastogenesis‐related genes expression

**Figure 6:**
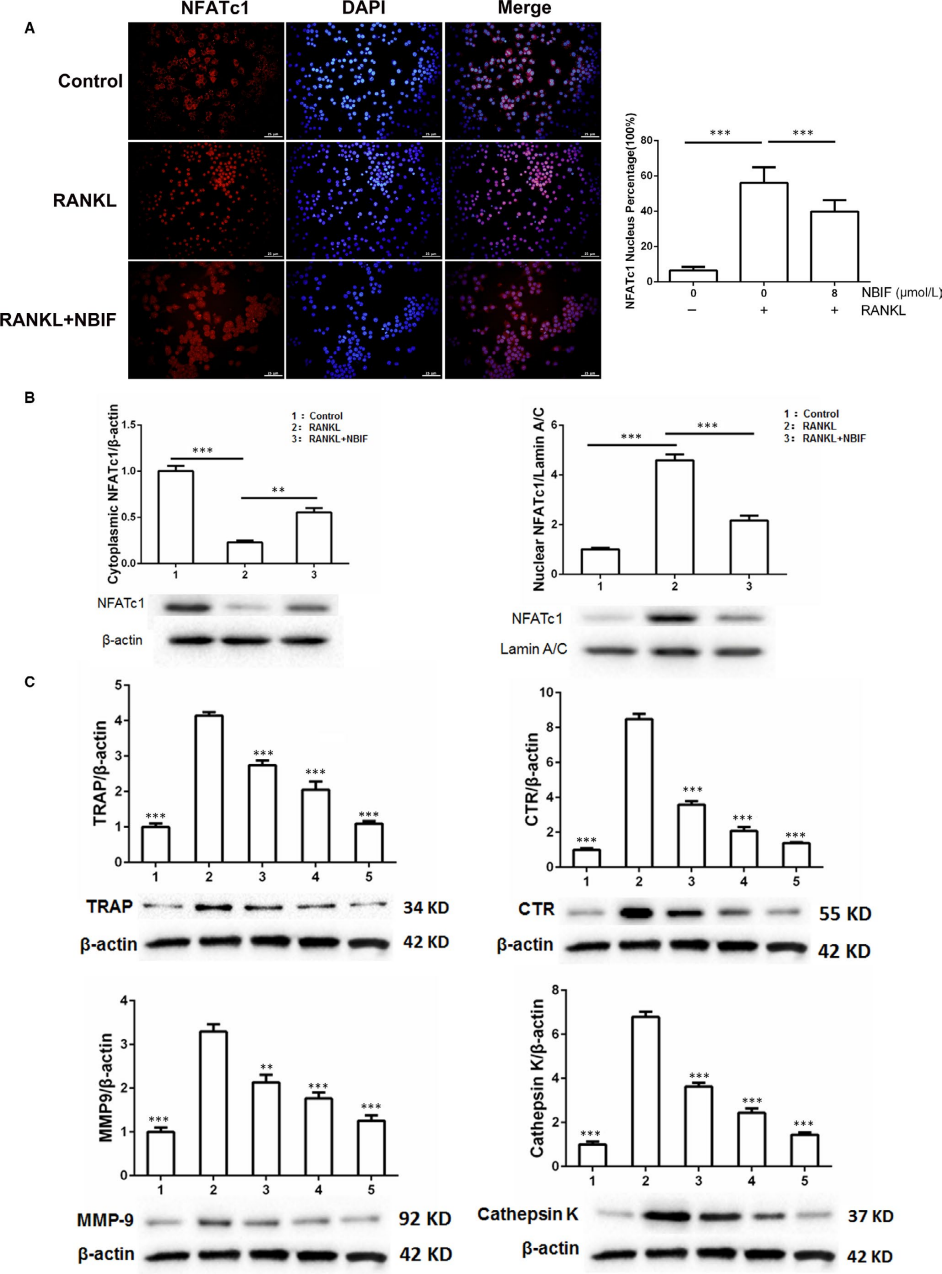
NBIF inhibits the nuclear translocation of NFATc1 and the expression of osteoclastogenesis‐associated marker genes. A, Representative immunofluorescent images and quantification of the nuclear translocation of NFATc1 in induced RAW 264.7 cells treated by NBIF. B, Western blot and band intensity analysis of the cytoplasmic and nuclear expressions of NFATc1 in the RANKL‐induced RAW264.7 cells treated by NBIF. C, Western blot and band intensity analysis of the expression of TRAP, CTR, MMP‐9 and Cathepsin K with β‐actin as the internal control (β‐actin blot images was reused in C for illustrative purposes). Groups are divided as follows: Group 1. RAW264.7 cells; Group 2. RAW264.7 cells induced with RANKL treated with PBS; Group 3. RAW264.7 cells induced with RANKL and treated with 2 μM NBIF for 7 days; Group 4. RAW264.7 cells induced with RANKL and treated with 4 μM NBIF for 7 days; Group 5. RAW264.7 cells induced with RANKL and treated with 8 μM NBIF for 7 days. (*P < .05, ***P < .001 vs. Group 2). Data are presented as mean ± SEM (n = 3)

It has been well established that the activation and nuclear translocation of NFATc1 plays a dominant role in transcriptional regulation of osteoclastogenesis marker genes (TRAP, CTR, MMP‐9 and Cathepsin K) which are correlated with osteoclast differentiation. To assess the effect of NBIF on RANKL‐induced activation of NFATc1, immunofluorescence staining was applied to determine the nuclear translocation of NFATc1. As immunofluorescence staining displayed, the translocation of NFATc1 from cytoplasm to nucleus was remarkably increased by RANKL, while NBIF significantly attenuated this RANKL‐mediated nuclear translocation of NFATc1 (Figure 6A and B). To investigate the role of NBIF in the expression of these aforementioned marker genes, RAW264.7 cells induced by RANKL were exposed to increasing concentration of NBIF (0, 2, 4 and 8 μM). Cells were then harvested for Western blot analysis at day 7 after the induction. We found that the expression of all these marker genes, during the period of RANKL‐induced osteoclastogenesis, was drastically decreased by NBIF treatments in a dose‐dependent way (Figure 6C). To sum up, the results implied that NBIF might exert inhibitory effects on osteoclastogenesis by suppressing osteoclastogenesis‐related genes expression.

### 3.11 NBIF attenuated bone loss by inhibiting osteoclast activation and promoting osteogenesis in ovariectomized mice

**Figure 7:**
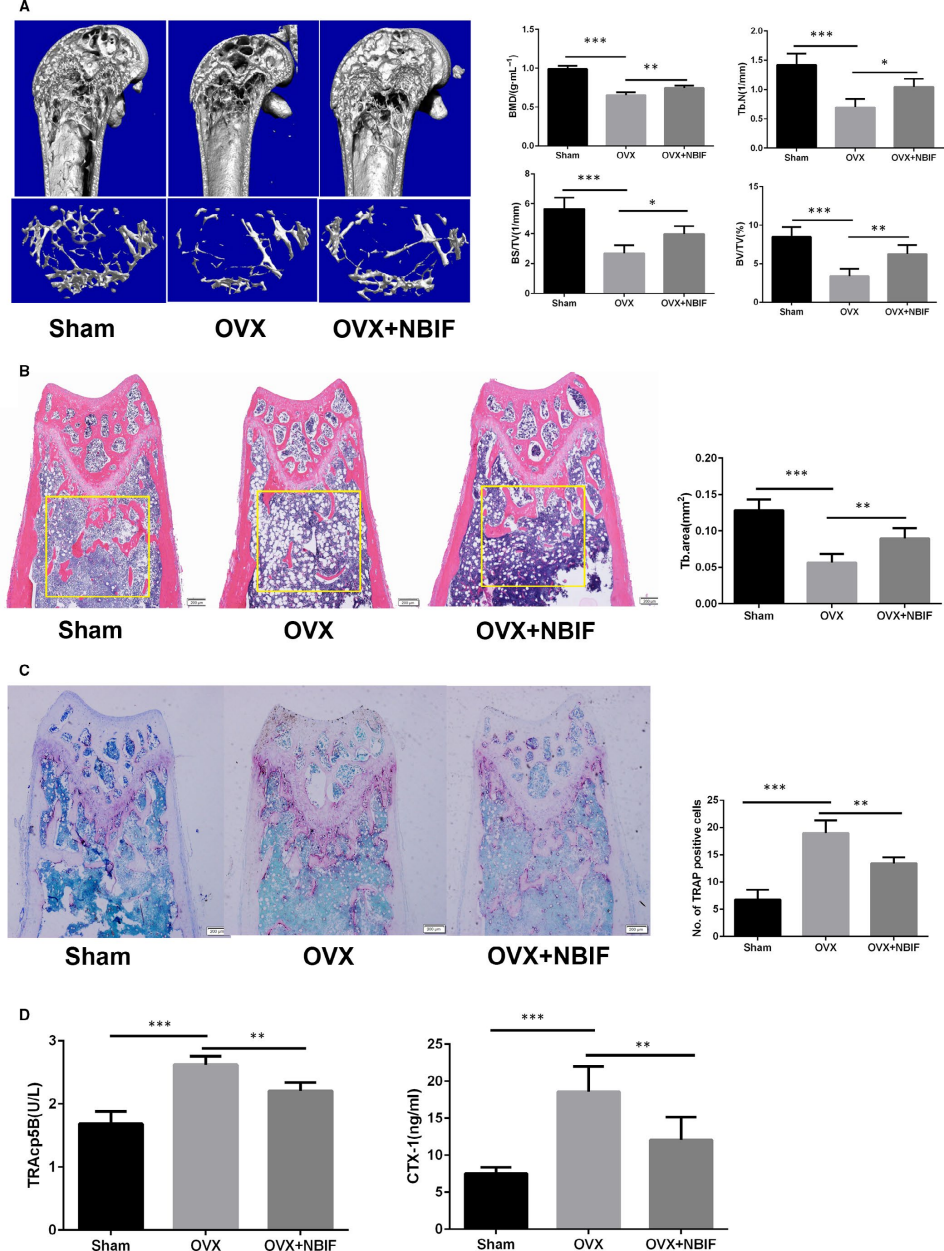
NBIF attenuates ovariectomy‐induced bone loss in vivo. A, Micro‐CT analysis of distal femurs from sham, OVX and OVX + NBIF group. Parameters quantification analysis: bone mineral density (BMD), trabecular number (Tb.N), bone surface area/total value (BS/TV) and bone value/total value (BV/TV). B, Representative H&E staining and quantification of the distal femoral trabecular area from each group 6 weeks after ovariectomy. C, Representative images of TRAP‐stained distal femoral sections and quantification of TRAP‐positive cells from each group. D, Analysis of TRAcp5B, CTX‐1 level in the serum by ELISA. (*P < .05, **P < .01, ***P < .001 vs. OVX group). Data are presented as mean ± SEM (n = 5)

To further validate the efficacy of NBIF in vivo, ovariectomy was performed in mice to mimic the pathological bone loss after the withdrawal of oestrogen. Micro‐CT showed that heavy trabecular bone loss was observed in the OVX group compared with that in the sham group, while NBIF treatment notably prevented the bone loss from ovariectomy as showing with the increased values of Tb.N, BMD, BS/TV and BV/TV in the 2‐ and 3‐dimensional photographs (Figure 7A). Compared with the OVX group, the NBIF group also illustrated higher bone mass maintenance presented by H&E staining images (Figure 7B). Furthermore, we carried out TRAP staining to determine whether osteoclasts were inhibited by NBIF intervention. The results displayed that the distal femur sections from ovariectomized mice were spread with increased TRAP‐positive multinucleated cells in the trabecular area, while fewer osteoclasts were found in the NBIF group than that in the OVX group (Figure 7C). Besides, the level of the serum CTX‐1 and TRAcp5B was measured to evaluate whether NBIF intervention ameliorated ovariectomy‐associated bone loss via restraining osteoclast activity. In comparison with the OVX group, remarkably decreasing serum levels of these two markers were detected in the NBIF group, reflecting the inhibitory effect of NBIF on osteoclasts’ function (Figure 7D). Surprisingly, the number of OCN‐positive osteoblasts and OCN serum level also saw a significant rise in the NBIF group in contrast to the OVX group, which indicated that NBIF also promoted osteogenesis in vivo (Figure S4A and B). Collectively, all these results suggested that NBIF attenuated OVX‐associated pathological bone loss by disturbing osteoclast activity and promoting osteogenesis.

## 4 DISCUSSION

**Figure 8:**
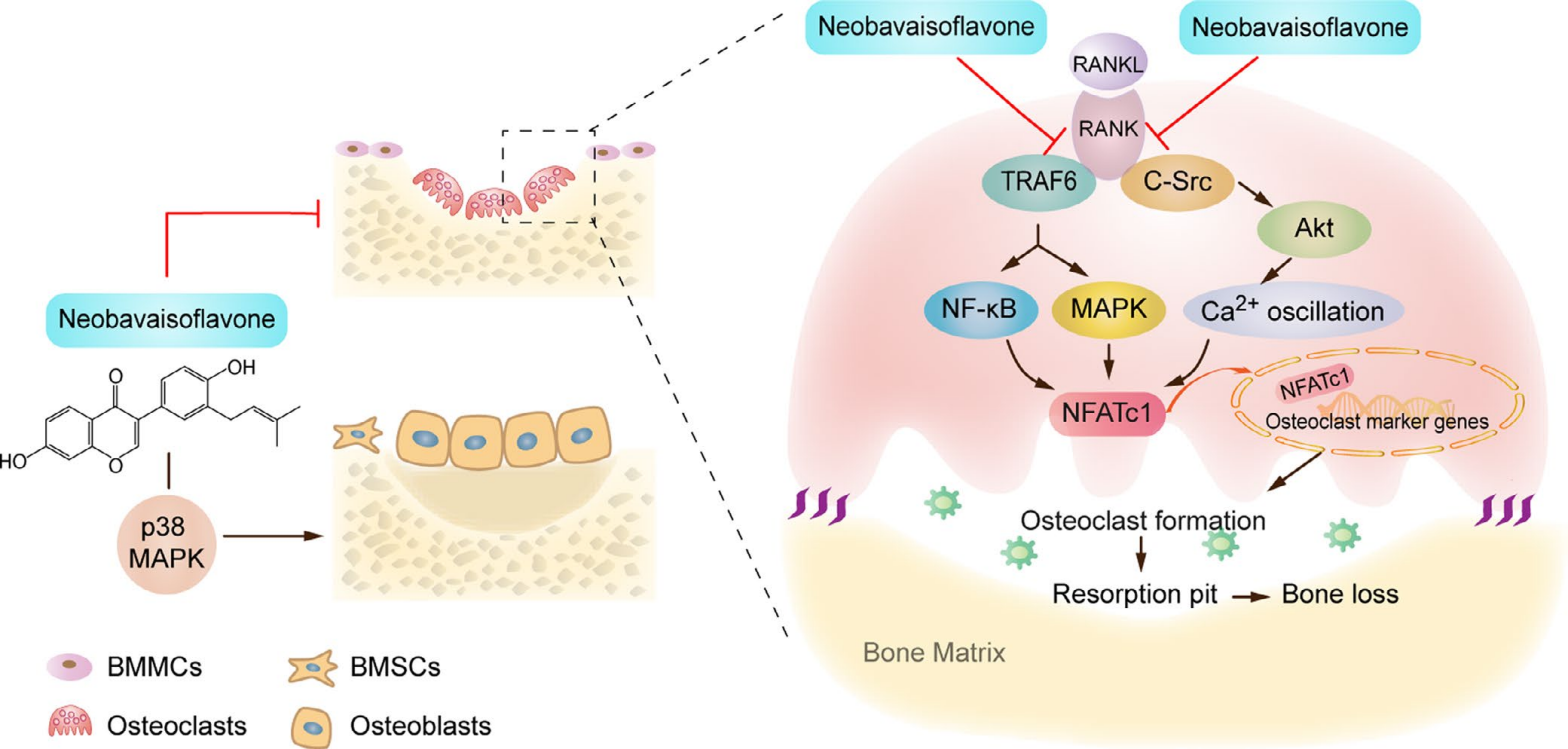
A brief diagram of the mechanism of NBIF inhibitory effects on RANKL‐mediated osteoclastogenesis

In this study, by the 2D RAW264.7/CMC/C18 column/TOFMS system, we discovered NBIF, a novel osteoclastogenesis‐suppressive compound extracted from *P corylifolia*. As far as we know, it is for the first time that NBIF is reported for its inhibitory effects on osteoclastogenesis in vitro and in vivo. In vitro, NBIF inhibited RANKL‐induced signalling pathways (MAPKs, NF‐κB and Akt) by blocking the interactions of RANK with TRAF6 and c‐Src. In vivo, NBIF protected against pathological bone loss induced by ovariectomy in mice by inhibiting osteoclastogenesis and promoting osteogenesis (Figure 8).

*P corylifolia* has been well‐known for centuries in China to possess medicinal values to accelerate fracture healing and strengthen the bone quality, especially for those PMOP women. However, the bioactive ingredients remain unclear. Currently, 2D CMC analytical system has been employed for detecting potential bioactive components from herbal extracts.^3^, ^4^ This system is based on the bioaffinity of bioactive components with the targeted cellular membranes. Murine monocytic cell line, RAW 264.7, generally accepted as the macrophage/pre‐OC population, differentiates into bone resorptive osteoclasts upon exposure to RANKL stimulation.^33^, ^34^ Therefore, in this study, we employed 2D RAW 264.7 cells CMC/C18 column/TOFMS system to explore potential bioactive components from *P corylifolia* for osteoclastogenesis regulation, and C_20_H_18_O_4_ was finally filtrated. Then, we resorted to Traditional Chinese Medicine and Chemical Composition Database to figure out the possible component. Eventually, we identified C_20_H_18_O_4_ as NBIF and speculated its potential osteoclastogenesis‐suppressive effects.

Dynamic bone formation and bone resorption always keep a synchronous balance for maintaining normal bone self‐renewal. Disturbance to this delicate balance by highly activated osteoclasts, particularly, leads to excessive bone resorption and impaired bone microstructure, having been proved to be responsible for various osteopenic disorders, including PMOP.^35^ As a result, inhibiting overactivated osteoclastogenesis could be an effective strategy to find a cure for pathological bone loss in these diseases.

In this study, we decided to explore the inhibitory effects of NBIF on osteoclastogenesis and its possible molecular mechanism. In vitro, we found little cytotoxic effects on RAW264.7 cells and BMMCs below 8 μM. As a result, 2, 4 and 8 μM were chosen for further in vitro experiments. We found that NBIF potently suppressed the differentiation of BMMCs and RAW 264.7 cells into mature osteoclasts induced by RANKL and M‐SCF at the early phase. Disrupted F‐actin formation and impaired pit‐formation activity indicated significant inhibitory effects on osteoclasts function because of NBIF treatments.

The process of osteoclastogenesis is mainly divided into two stages. The first one is M‐CSF‐induced survival maintenance, the proliferation, differentiation of BMMCs, and the second is RANKL‐induced maturation and differentiation into osteoclasts. M‐CSF is a well‐recognized vital factor participating in the proliferation and differentiation of BMMCs and osteoclast precursors through binding to its receptor, c‐Fms. Particularly, M‐CSF acting via c‐Fms induces the expression of RANK, which is indispensable for RANKL/RANK signalling activation, and is therefore essential for the proliferation and differentiation of osteoclast precursors.^13^ Here, we found little effects of NBIF on M‐CSF‐induced proliferation of BMMCs, without inhibition of c‐Fms and RANK expression which indicated NBIF might exert its inhibitory effects on osteoclastogenesis through RANKL/RANK signalling, instead of interrupting M‐CSF‐induced proliferation and differentiation of BMMCs and pre‐osteoclasts. RANKL/RANK signalling pathway leads the central role of osteoclasts differentiation and maturation, and the blockade of RANKL/RANK signalling is now proved to be an effective strategy to prevent and ameliorate bone loss in PMOP.^36^ When RANKL conjugates to activate the RANK receptor, TRAF6 and c‐Src are both recruited, activating the downstream signalling cascades,^19^, ^37^, ^38^ including NF‐κB, MAPKs and Akt signalling pathways. RANK‐TRAF6 interaction activated NF‐κB and MAPKs pathways, through phosphorylation of IκB, P50, P65, ERK, JNK and P38. RANK‐c‐Src interaction leads to the activation of the Akt signalling pathway, including the phosphorylation of Akt, which subsequently triggered calcium oscillation.^21^ Finally, activation of NF‐κB, MAPKs and Akt pathways provoked the activation and translocation of NFATc1, the master transcription factor that regulates the expression of osteoclastogenesis‐associated marker genes, TRAP, CTR, MMP‐9, Cathepsin K included. Here, we demonstrated that both RANK‐TRAF6 interaction and RANK‐c‐Src interaction induced by RANKL were simultaneously disrupted in the presence of NBIF. Therefore, downstream pathways including NF‐κB, MAPKs and Akt pathways were found to be inhibited by NBIF. Following the inactivation of Akt signalling, cytoplasmic calcium level fluctuation was detected to be suppressed by NBIF intervention. Subsequently, the activation and translocation of NFATc1 were disrupted. Osteoclastogenesis‐related markers genes (TRAP, CTR, MMP‐9 and Cathepsin K) were also down‐regulated after the exposure to NBIF treatments. To sum up, these results demonstrated that NBIF inhibited osteoclastogenesis and function through blocking RANKL‐induced TRAF6 and c‐Src recruitment, NF‐κB, MAPKs and Akt pathways activation, calcium oscillation and NFATc1 translocation.

With regard to the pathogenesis of PMOP, it is caused by the abnormal coupling of bone remodelling disturbed by unbalanced bone resorption and bone formation. Withdrawal of oestrogen leads to increased osteoclastogenesis and enhanced bone resorption accompanied by substantial bone loss.^39^, ^40^ Therefore, targeting overactivated osteoclasts provides an effective option for the treatment of PMOP. Overactivated osteoclasts contribute to excessive bone resorption in the pathological physiology of PMOP. We further confirmed our in vitro study results using ovariectomized mice model to mimic the PMOP pathological state. Before we started, we tested the maximum dosage of NBIF given per day for consecutive 7 days, and 30 mg/kg/day was found to be the maximum dosage which was used to explore in vivo effects of NBIF. We discovered that NBIF significantly rescued bone loss after ovariectomy in mice, as demonstrated by H&E staining and microcomputed tomography of the distal femurs. For TRAP staining, we found that the number of TRAP‐positive mature osteoclasts drastically reduced around the trabecula. Serum levels of TRAcp5B and CTX‐1 which represented the bone resorption level in vivo were also consistently reduced. In short, NBIF inhibited osteoclastogenesis in vivo and prevented bone loss of ovariectomy‐induced bone loss.

Targeting the inadequate or impaired bone formation should be another promising strategy for pathological bone loss and defective bone repair.^41^, ^42^ NBIF was previously reported to be capable of inducing osteogenesis, stimulating bone matrix proteins expression and mineralization in vitro, mainly through the P38‐mediated up‐regulation of transcriptional factors and osteoid genes.^31^ Application of P38 inhibitor abrogated the osteogenesis‐stimulating effects induced by NBIF on the expression of osteogenic marker genes (Runx2, Osx). Consistent with this finding, our study discovered that NBIF also promoted osteogenesis in vivo, presented by more OCN‐positive mature osteoblasts in the distal femurs and higher OCN level in the serum. It is well known that the bone remodelling process pathologically accelerates by ovariectomy‐associated oestrogen deficiency. Under this circumstance, overactivated osteoclastogenesis is insufficient to couple equivalent bone formation by osteoblasts at a period of a single remodelling cycle, resulting in a net bone loss.^43^ Intriguingly, our results discovered that NBIF rescued ovariectomy‐induced bone loss and acted to correct the unbalanced bone coupling disorders by inhibiting osteoclastogenesis and promoting osteogenesis simultaneously. However, the molecular targets of NBIF with regard to osteoclastogenesis still need to be clarified in the future.

In summary, 2D RAW 264.7 cells CMC/C18 column/TOFMS system is useful in discovering potential natural products from traditional Chinese medicine. With the exploitation of this system, we explored NBIF and validated that NBIF might serve as an osteoclastogenesis inhibitor and also as an osteogenetic inducer at the same time, for the treatment of PMOP. As such, NBIF would be a promising and useful medical candidate for the treatment of osteoporosis and other osteolytic bone diseases.

## CONFLICTS OF INTEREST

The authors declare that there is no conflict of interest regarding the publication of this article.

## AUTHOR CONTRIBUTION

**Huiwen Chen:** Conceptualization (equal); Investigation (equal); Writing‐original draft (equal). **Chao Fang:** Investigation (equal); Writing‐original draft (equal). **Xin Zhi:** Conceptualization (equal); Investigation (equal); Writing‐original draft (equal). **Shaojun Song:** Supervision (supporting); Validation (equal); Writing‐review & editing (equal). **Yanqiu Gu:** Methodology (supporting). **Xiaofei Chen:** Methodology (supporting). **Jin Cui:** Data curation (supporting); Validation (supporting). **Yan Hu:** Data curation (supporting); Validation (supporting). **Weizong Weng:** Formal analysis (supporting). **Qirong Zhou:** Formal analysis (equal). **Yajun Wang:** Formal analysis (equal). **Yao Wang:** Formal analysis (equal). **Hao Jiang:** Formal analysis (equal). **Xiaoqun Li:** Formal analysis (equal). **Liehu Cao:** Writing‐review & editing (supporting). **Xiao Chen:** Conceptualization (supporting); Writing‐original draft (supporting); Writing‐review & editing (supporting). **Jiacan Su:** Project administration (lead); Supervision (lead); Writing‐review & editing (lead).

## Supporting information

Fig S1

Fig S2

Fig S3

Fig S4

## Data Availability

Data used to support the findings of this study are available from the corresponding authors upon request.
