# Supplementary figures and images for: Neobavaisoflavone inhibits osteoclastogenesis through blocking RANKL signalling‐mediated TRAF6 and c‐Src recruitment and NF‐κB, MAPK and Akt pathways
Source: J Cell Mol Med. 2020 Jun 30;24(16):9067–84. doi: 10.1111/jcmm.15543 (PMC7417698; doi:10.1111/jcmm.15543)

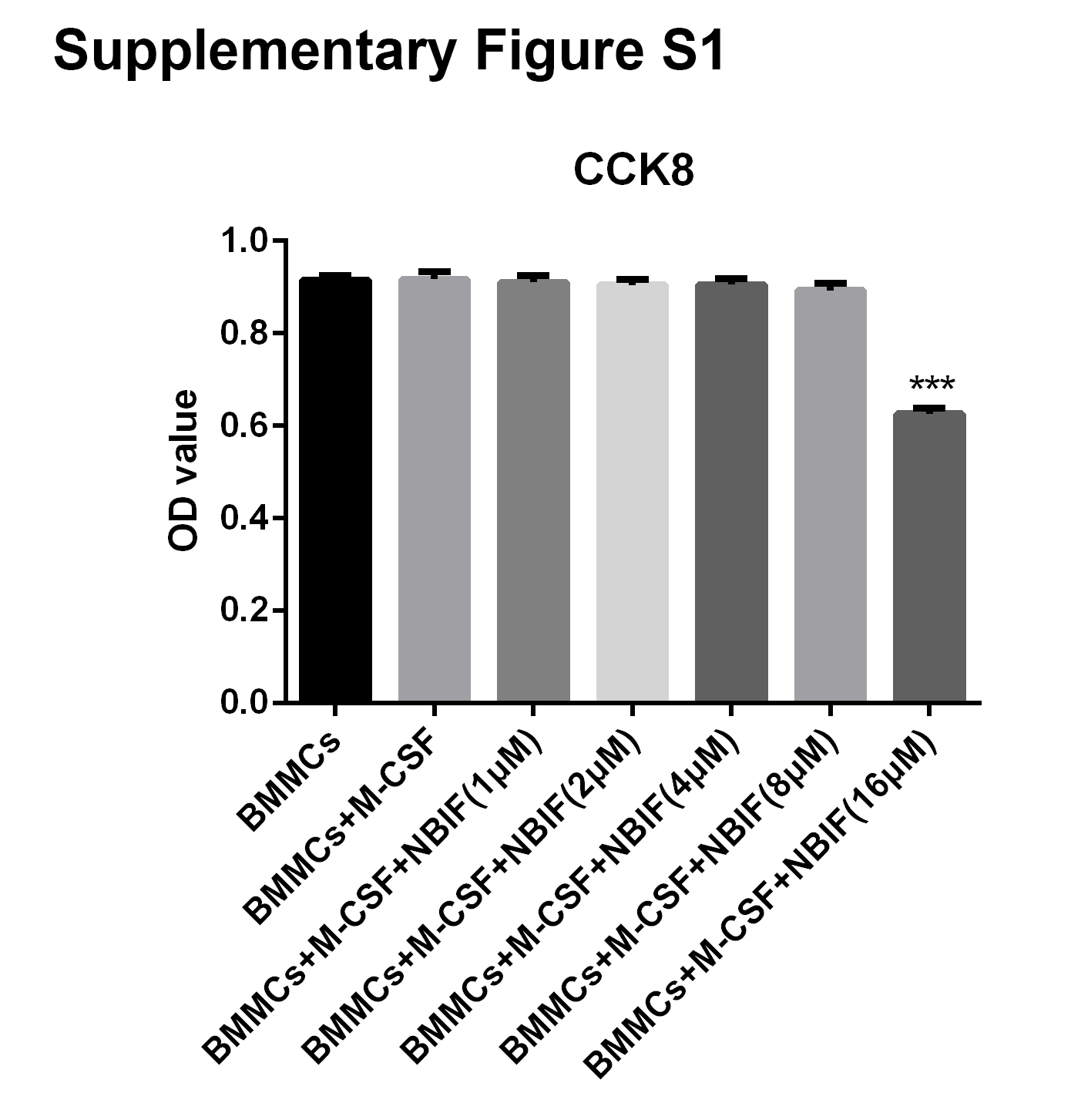

Supplement: Supplementary file 1 — Fig S1 [file JCMM-24-9067-s001.tif]

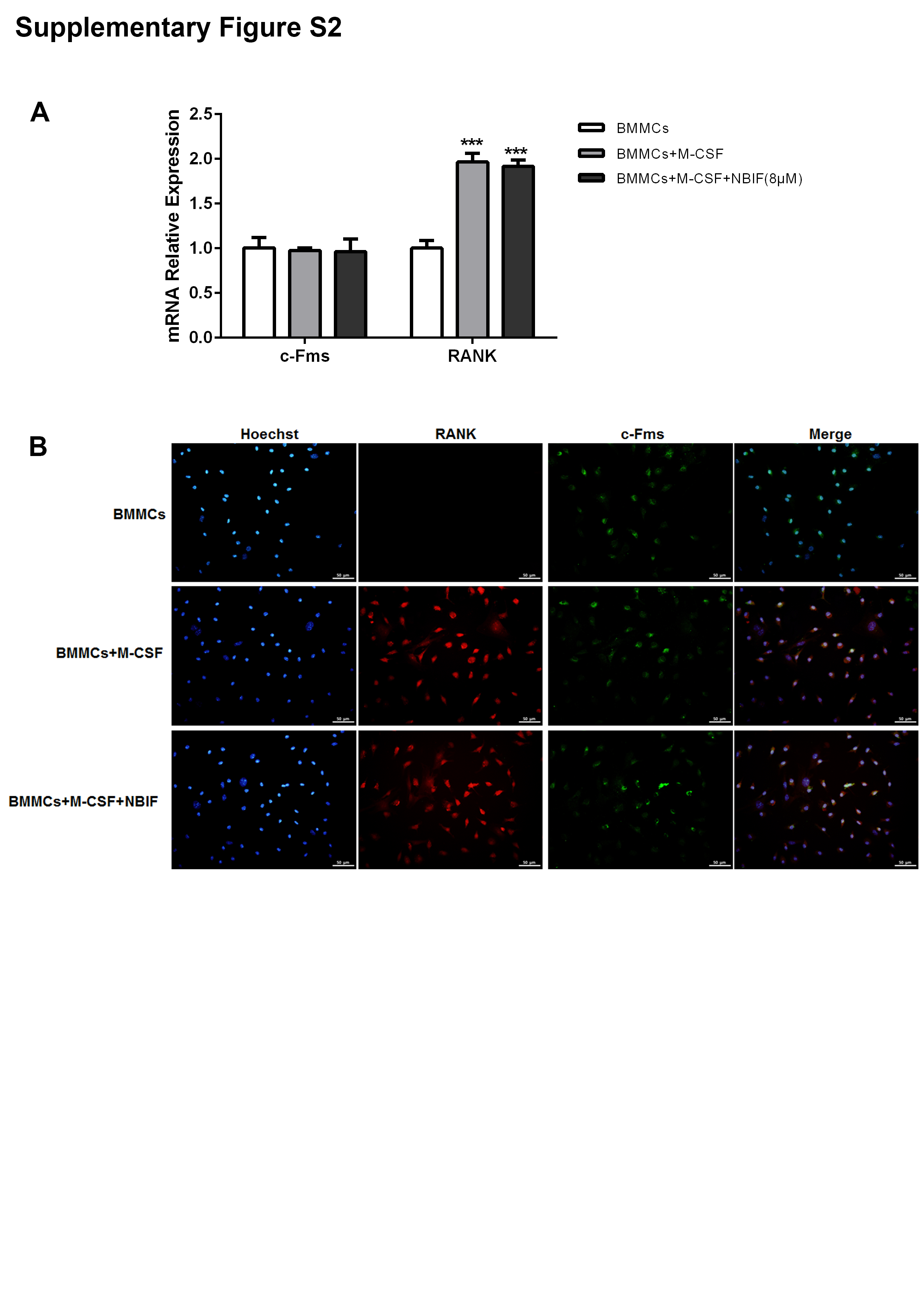

Supplement: Supplementary file 2 — Fig S2 [file JCMM-24-9067-s002.tif]

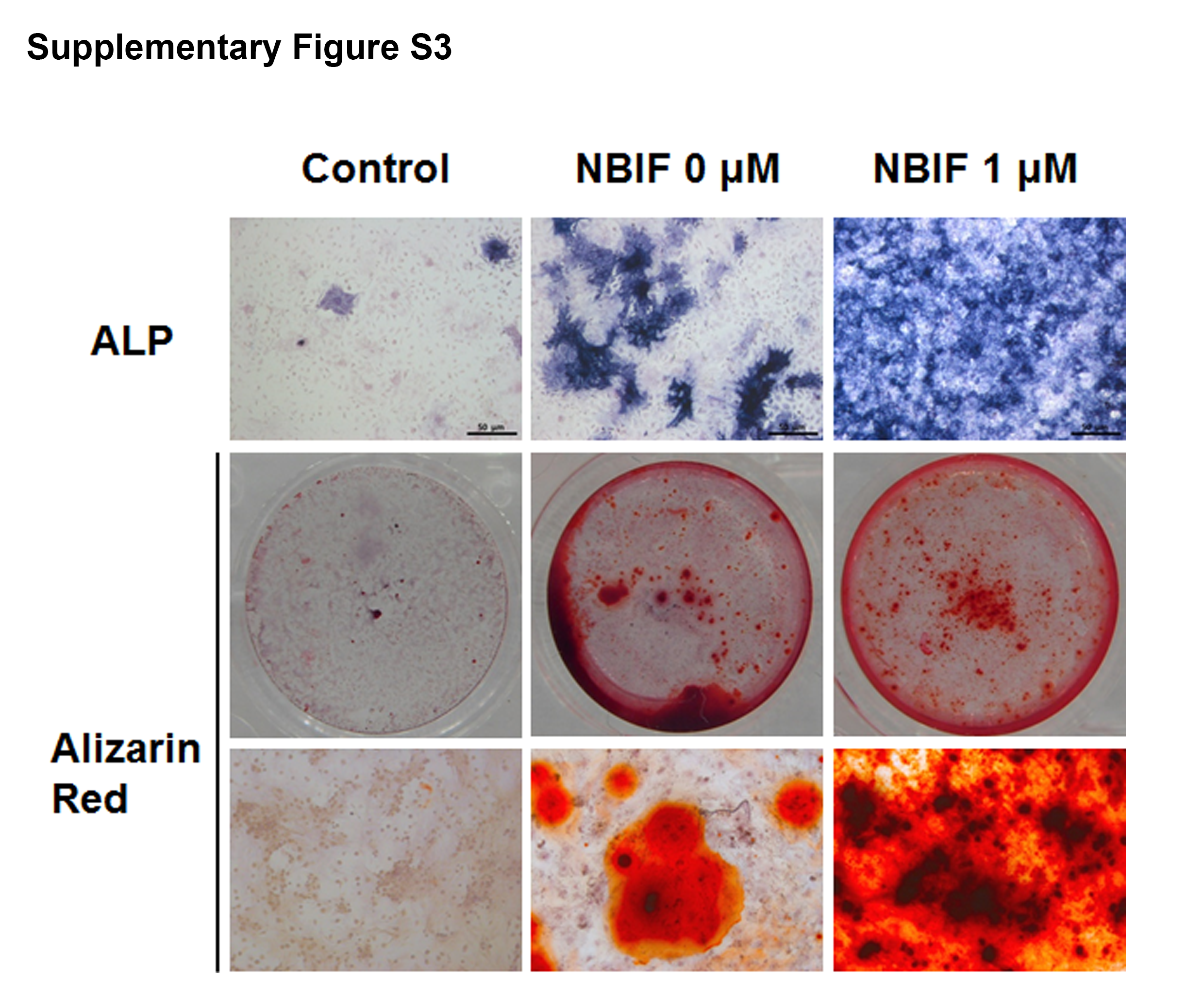

Supplement: Supplementary file 3 — Fig S3 [file JCMM-24-9067-s003.tif]

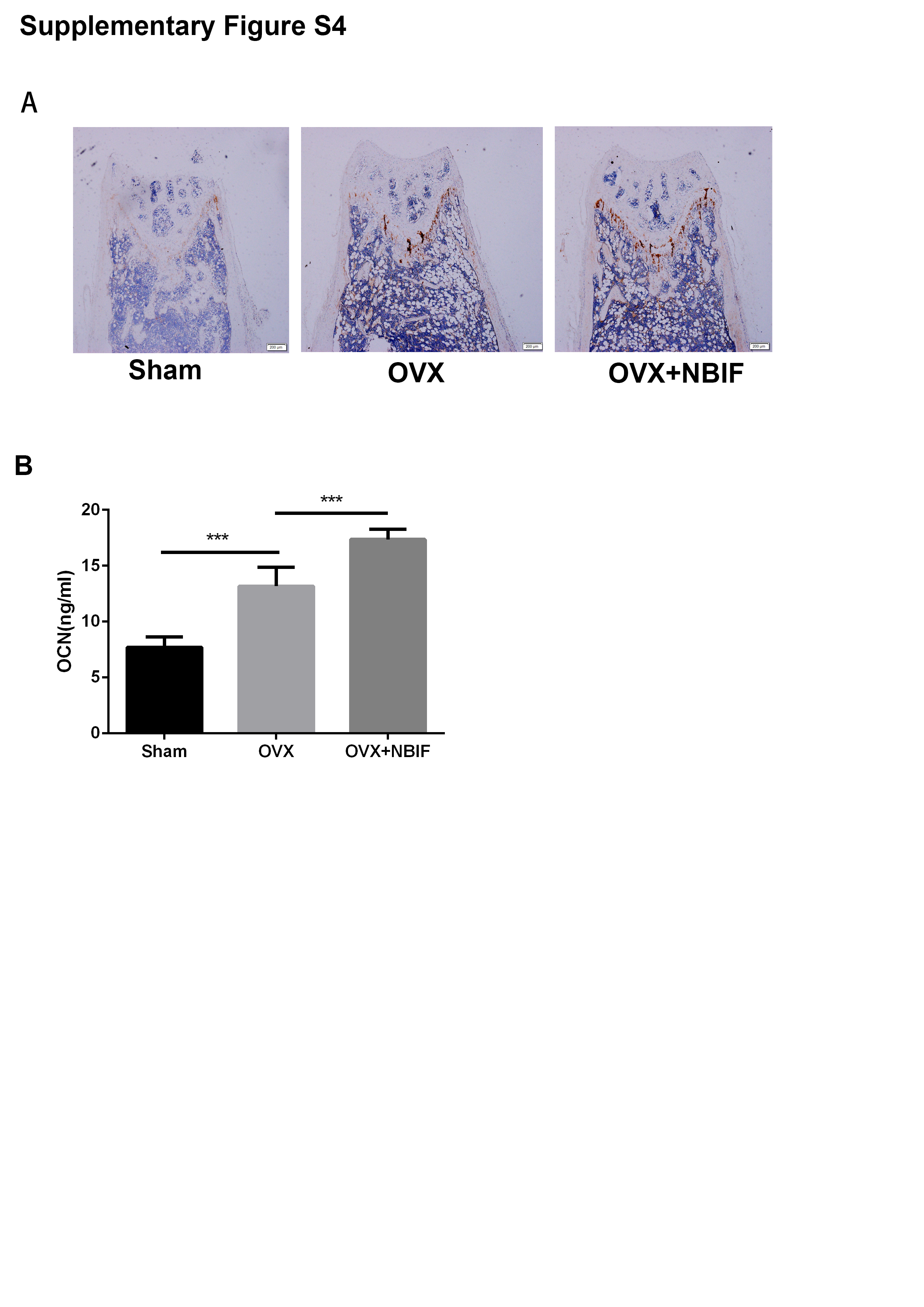

Supplement: Supplementary file 4 — Fig S4 [file JCMM-24-9067-s004.tif]
